# Polyelectrolyte Complexes from Oppositely Charged Filamentous Viruses

**DOI:** 10.1021/acs.langmuir.2c02790

**Published:** 2023-03-22

**Authors:** Hanna Anop, Johan Buitenhuis

**Affiliations:** † Forschungszentrum Jülich, IBI-4, Biomacromolecular Systems and Processes, 52425 Jülich, Germany; ‡ Cordouan Technologies, Cité de la Photonique, 11 Avenue Canteranne, 33600 Pessac, France

## Abstract

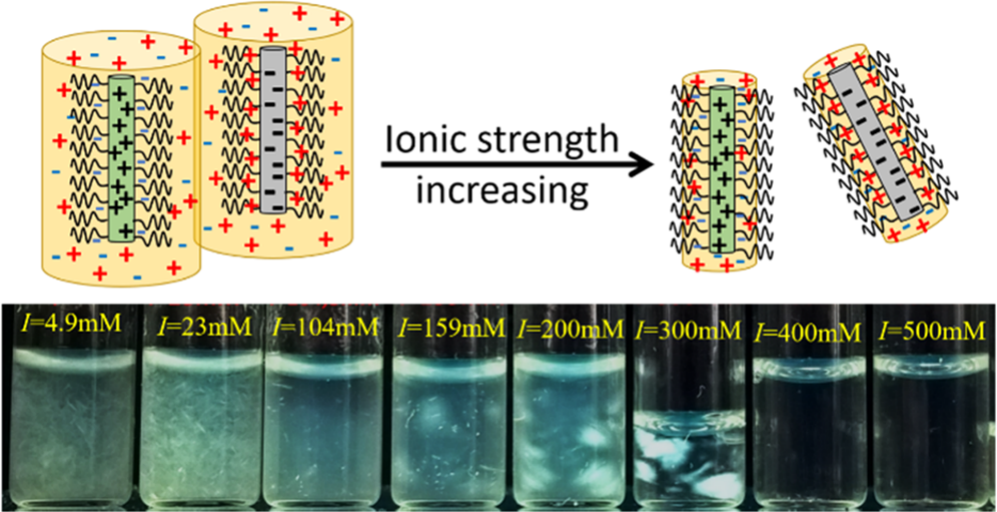

Here, we present an explorative study on a new type of polyelectrolyte complex made from chemically modified filamentous fd viruses. The fd virus is a semiflexible rod-shaped bacteriophage with a length of 880 nm and a diameter of 6.6 nm, which has been widely used as a well-defined model system of colloidal rods to investigate phase, flow, and other behavior. Here, chemically modified viruses have been prepared to obtain two types with opposite electrical charges in addition to a steric stabilization layer by poly(ethylene glycol) (PEG) grafting. The complex formation of stoichiometric mixtures of these oppositely charged viruses is studied as a function of virus and salt concentration. Furthermore, static light scattering measurements show a varying, strong increase in scattering intensity in some samples without visual macroscopic complex formation. Finally, the results of the complex formation are rationalized by comparing to model calculations on the pair interaction potential between oppositely charged viruses.

## Introduction

Mixing solutions of oppositely charged polyelectrolytes (PEs) may lead to complex formation, or coacervation, between the polycation and the polyanion. These polyelectrolyte complexes (PECs) are of interest for a range of (potential) applications, like tailoring of wood fiber surfaces, flocculation applications, aggregation of food proteins, DNA and polycations as gene delivery vectors, and pharmaceutical applications of polyelectrolyte complex nanoparticles (reviews are given in refs (1−5)). In addition, fundamental studies have been performed yielding basic principles on the formation and properties of polyelectrolyte complexes in relation to their composition (reviews given in refs (3−7)), which is useful to direct the development of new complexes with tuned properties.

In a simple model consideration, the formation of a polyelectrolyte complex (PEC) can be described with the following reaction^3^
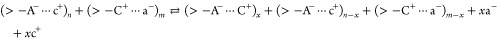
with >−A^–^ and >−C^+^ being the charged monomeric units of the polyelectrolyte and a^–^ and c^+^ are small salt ions that function partly as counterions. The three dots ··· denote the electrostatic interaction between two opposite charges, either between two monomeric units denoted as (>−A^–^···C^+^) or between a monomeric unit and a counterion denoted as (>−A^–^···c^+^) or (>−C^+^···a^–^). This simple model already demonstrates that an increase in salt concentration drives the PEC formation reaction back, i.e., salt counteracts PEC formation, as is also found experimentally. In a bit more detail, the formation of PECs is driven to a large part by the gain in entropy from the release of the low-molecular-weight counterions into the bulk solution. At high salt concentrations, complexes can dissolve completely. Typically, (initial) complex formation is fast and is (close to) diffusion-controlled; however, nonequilibrium structures can easily be formed, as reflected by the use of the term scrambled egg structure for disordered PEC structures.^2−4^ In terms of the reaction given above, in nonequilibrium structures, part of the charged groups in the PEs might still be interacting with the low-molecular-weight ions. The first approximate theory in which a phase separation in a PE-rich and a PE-depleted phase can be calculated was given by Voorn and Overbeek,^8^ and still recent experimental results on phase diagrams are successfully compared to this theory.^9^ Nevertheless, if detailed specific interactions in experimental systems become important, a complete model description becomes difficult, even for more extended theories.^6,10^ In particular, it also becomes difficult to differentiate between specific effects and more general effects. From an experimental point of view, general effects and trends can be studied with well-defined model systems. Here, we present a new model system for PEC formation, where specific interactions are expected to be of minor importance.

The system is based on chemical modifications of a filamentous bacteriophage, the so-called fd virus, which can be grown by replication within certain types of nonpathogenic *Escherichia coli* bacteria. It consists of a single-stranded circular DNA molecule with a coat of 2700 major coat proteins and a small number of minor coat proteins at the ends. The fd virus is a highly negatively charged particle^11^ with a length of 880 nm, a width of 6.6 nm, and a persistence length of 2.2 μm,^12^ so that it can be considered a semiflexible rod. Therefore, for increasingly concentrated fd solutions, a transition from an isotropic solution to a nematic solution can be observed, where the concentration at which the transition takes place depends on the salt concentration in the solution.^13^ Due to its unique properties in combination with its simple and reproducible production procedure, the fd virus has been used many times as an almost ideal model system in fundamental studies on the properties of colloidal dispersions of filamentous or cylindrical particles.^12−14^ For the same reasons, here, we use the fd virus as a well-defined filamentous material, for which the biological properties are only of interest in their production/growth.

**Figure 1:**
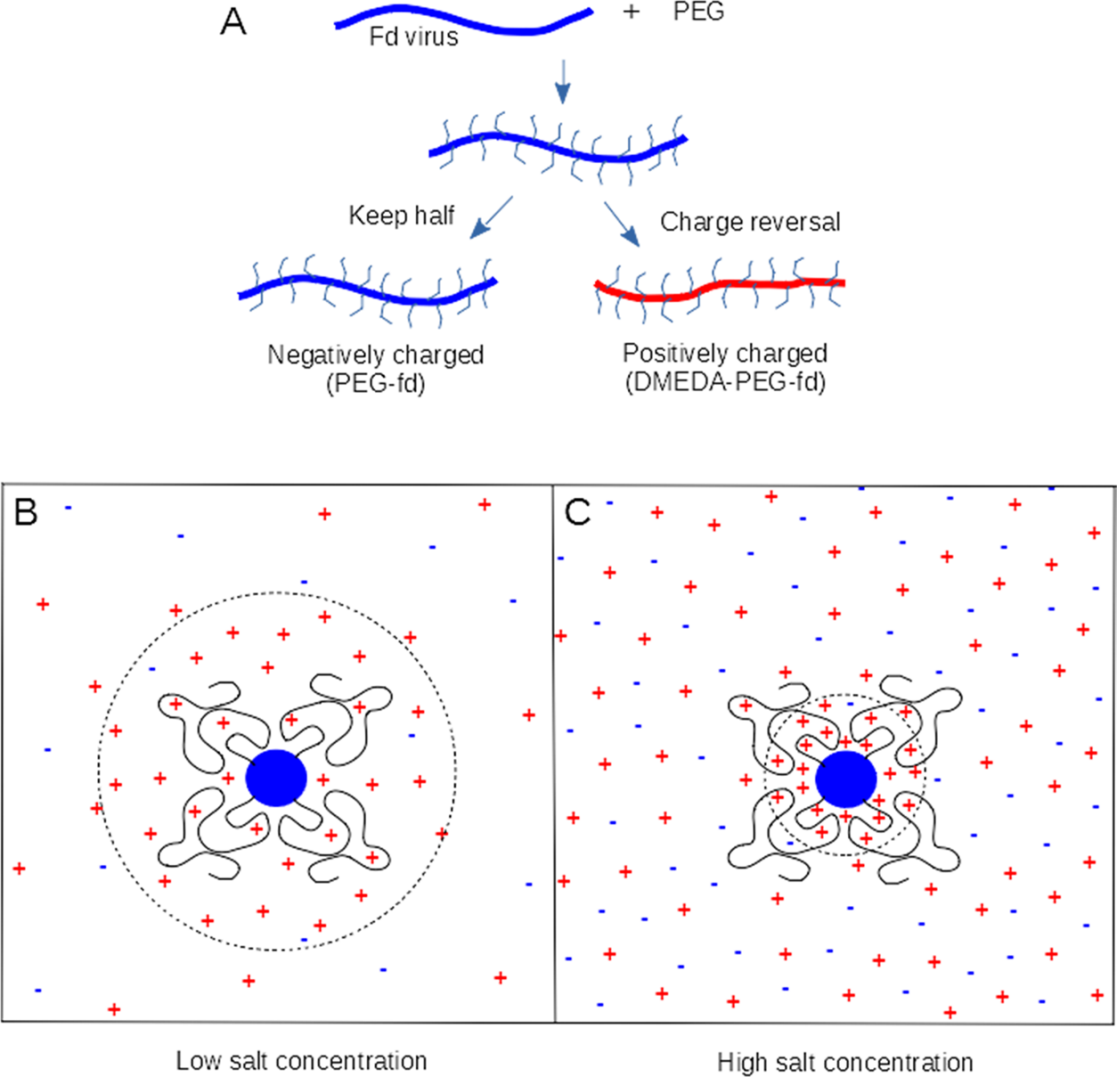
(A) Schematic overview of the synthesis procedure and (B,C) artists’ impression of the cross section of PEG-fd. The color blue denotes negative charges and red denotes positive charges, and the black lines denote PEG chains. Note that the dimensions are not to scale. For low salt (B), the electrical double layer extends beyond the PEG steric stabilization layer, whereas for high salt (C), the electrical double layer remains within the PEG steric stabilization layer. A dotted circle indicates the extent of the electrical double layer.

In this study, the fd viruses are grafted with poly(ethylene glycol) (PEG) to add a steric stabilization layer to the viruses,^13^ after which for half of the PEG-grafted particles, the charge (at neutral pH) is reversed using carbodiimide chemistry to bind *N*,*N*-dimethylethylenediamine (DMEDA) on the solvent-exposed carboxyl groups on the surface of the fd virus,^15^ as illustrated in Figure 1A. Mixtures of these positively and negatively charged bottle-brush-shaped viruses are expected to form polyelectrolyte complexes depending on the virus concentration and ionic strength. If the salt concentration is high enough, so that the electrostatic double layer around the viruses is fully inside the PEG layer (see Figure 1C), the oppositely charged viruses are expected not to “feel” each other’s charge, so that no complex formation is expected, whereas for lower salt concentrations, the electrostatic double layer will be partially outside the PEG layer (see Figure 1B), resulting in an electrostatic attraction between oppositely charged viruses, which might result in complex formation.

A limited number of studies have been performed on the PEC formation of other cylindrical bottle brush polymers, often as a combination of a bottle brush polymer with an oppositely charged linear PE.^16^ As far as we know, only two publications have considered PEC formation between oppositely charged cylindrical brush polymers. Duschner et al.^17^ studied the PEC formation between a cylindrical bottle brush/surfactant complex and a polymer bottle brush and tuned the charge on the polymer brush and compared structures formed with a highly as well as a slightly charged brush. Interestingly, with the highly charged brush, kinetically controlled PEC structures were observed, whereas the slightly charged brush resulted in topologically controlled PECs. A second study on PEC formation between two oppositely charged polymer brushes, published by Raguzin et al.,^18^ only found kinetically controlled PECs, described as scrambled egg structures. Already these two studies suggest a tendency toward kinetically controlled structures, where equilibrium structures seem to require careful tuning of a small effective attraction between the oppositely charged polymer brushes. A comparison of these aspects to the present study will be made.

To the best of our knowledge, PEC formation between oppositely charged cylindrical bottle brushes, both with noncharged side chains (“hairs”) to limit direct charge interactions,^19^ has not been published. Special for our virus-based polyelectrolyte complexes is the combination of the electrostatic attraction with a steric repulsive interaction between stiff viruses, which allows for model calculations on the effective interaction between two oppositely charged viruses. Furthermore, the filamentous shape of the viruses could result in the formation of liquid crystalline phases, whereas for different composition gels, flocculation or phase separations or transitions might be observed. As the number of variables is large, the present explorative study focuses on the effect of the overall virus concentration and the ionic strength, i.e., stoichiometric (1:1) complexes are considered.

This paper is organized as follows. In the next section, the method to calculate the interaction between two oppositely charged viruses, modeled as two oppositely charged cylinders, including salt and pH dependence, is described. The Experimental Section provides details about sample preparation and measurements. The Results and Discussion section explores the PEC formation of the oppositely charged fd viruses and is divided into three parts, describing (1) a state diagram showing the state of PEC formation or not as a function of virus and salt concentration, (2) static light scattering on possible small PEC aggregates or other structures formed in visually clear solutions, and (3) theoretical calculations on the interaction between two oppositely charged viruses, discussed in the context of the experimental results described in parts 1 and 2. Finally, the main findings in this study are summarized in the Conclusions section.

## Calculation of the Interaction between Two Oppositely Charged Viruses

Two interacting oppositely charged viruses are modeled as two smooth charged cylinders with a high aspect ratio, where the pair interaction potential is a function of the distance as well as the angle between the cylinders, the ionic strength, and the pH of the sample. In these calculations, the effect of the PEG steric stabilization layer is added by introducing a distance of the closest approach *H*, which is the only adjustable parameter in the calculations. It is assumed that the PEG grafting does not significantly change the charge as compared to the bare virus. In addition, for the oppositely charged particles, the absolute positive and negative charges are assumed to be equal. This is legitimized by the experimental pH being adjusted to approach this point, which is based on electrophoretic measurements as a function of pH (see Results and Discussion section, Figure 3). Therefore, only the negative charge is calculated and the positive charge is assumed to have the same absolute value.

**Figure 2:**
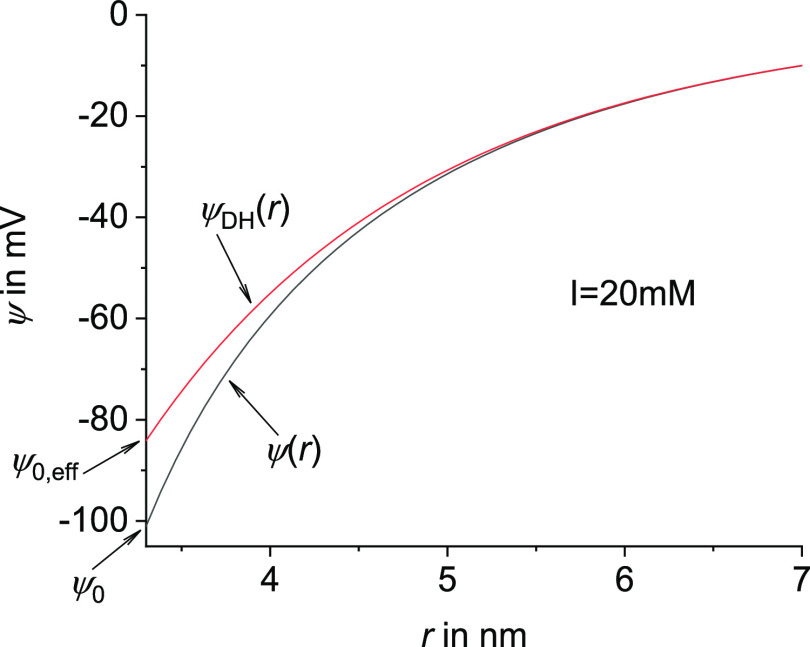
Example of the potential distribution from eq 8 based on the full Poison–Boltzmann equation, compared to the curve using the Debye–Hückel approximation (eq 10) with an effective surface potential such that the curves coincide at higher values of *r*. The difference between the two curves is described by multiplying by the function γ(ψ(*r*), κ*r*), which is one for a larger *r* when the two curves coincide. Note that the curves start at the surface of the virus/cylinder, at *r* = 3.3 nm, which is equal to the radius of the virus *a*.

The electrostatic pair interaction between two rods of a large aspect ratio is described following Brenner and Parsegian^20^ adding a correction for their eqs 17 and 18 for a numerical error of a factor two missing as described by Stigter.^21^ We note that these two publications^20,21^ use the older unrationalized units; here, we use the current more common rationalized units. The description of the interactions is valid for all distances between the cylinders for which a dividing surface can be defined, where the potential can be written as a linear superposition of the single cylinder potentials (i.e., low potentials in the overlap region). Therefore, the surface potentials of the individual rods may be much larger than the potential in the overlap region because the steric repulsion by the PEG layer limits the distance of the closest approach. For parallel cylinders, the interaction potential is given by^20,21^
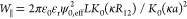
1where ε_0_ is the vacuum permittivity, ε_r_ is the relative dielectric constant of the solvent, ψ_0,eff_ is the effective Debye–Hückel surface potential that is chosen such that its long-distance Debye–Hückel potential distribution ψ_DH_(*r*) coincides with that of the potential ψ(*r*) obtained from the full nonlinear Poisson–Boltzmann theory (see Figure 2), *L* is the length of the cylinder, *a* is the radius of the rods, *K*_0_ is the zeroth-order modified Bessel function of the second kind, *R*_12_ is the distance between the center axis of the cylinders, and 1/κ is the Debye length of the medium, calculated from
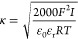
2where *F* is the Faraday constant, *R* is the molar gas constant, *T* is the absolute temperature, and *I* is the ionic strength in mol dm^–3^, which for monovalent ions is given by , where *c_i_* is the concentration of each ion *i* in mol dm^–3^. Equation 1 is a good approximation if the minimum distance between the surfaces of the rods (*R*_12_ – 2*a*) is larger than 1/κ.^20^ For cylinders with a parallel orientation but a shift of the two centers, it might be a reasonable approximation to substitute *L* with the length over which the cylinders are next to each other. For tilted cylinders, the interaction potential is given by^20,21^
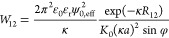
3where φ is the tilt angle between the rods and *R*_12_ is the shortest distance between the center axis of the cylinders. Equation 3 is accurate for (*R*_12_ – 2*a*) > 1/κ and sin φ ≫ 2(*a* + 1/κ)/*L*.

The description of the electrostatic surface potential ψ_0_ of the cylinders within the nonlinear Poisson–Boltzmann theory is following Stigter^22^ and is applied to the fd virus as described by Buitenhuis^23^ to obtain the surface potential and potential distribution as a function of pH and the ionic strength, also called a regulated charge description. Considering the dissociation constants of the seven solvent-exposed ionizable groups per coat protein, with an expected p*K*_a_ of 4.5 for two glutamate and three aspartate groups and 7.9 for one terminal amino group and 10.1 for one lysine group per coat protein, and 2700 major coat proteins on the surface of the fd virus, the charge on the virus *Q* is given by^23^
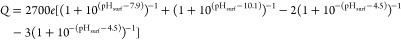
4with pH_surf_ being the pH (=–log[H^+^]) at the surface of the virus and *e* being the elementary charge. The surface pH is related to the bulk pH (pH_bulk_) by
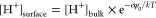
5where *k* is the Boltzmann constant, *T* is the absolute temperature, and ψ_0_ is the electrostatic surface potential obtained from nonlinear Poisson–Boltzmann theory given by^22,23^
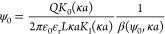
6where *K*_1_ is the first-order modified Bessel function of the second kind and β is a correction factor between the result from the linearized to the full Poisson–Boltzmann equation, i.e., for β = 1, the Debye–Hückel result from the linearized Poison–Boltzmann equation is obtained. The correction factor β was systematically calculated and tabulated by Stigter,^22^ and an empirical function was fitted to these values by Buitenhuis, from which β can be calculated as^23^
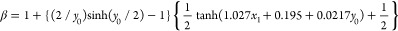
7where *x*_1_ = ^10^log(κ*a*) and *y*_0_ = ψ_0_*e*/*kT* is the reduced surface potential. Equation 7 is valid for *y*_0_ values up to 8. Now, *Q* and ψ_0_ can be calculated as a function of *I* and pH_bulk_, by simultaneously solving eqs 4–7, which has to be done numerically. One possibility to do this is using trial surface potentials ψ_0_ and calculating the surface charge from eqs 4 and 5 and also from eqs 6 and 7. If these two surface charges are equal, that charge and the corresponding surface potential are the results for *Q* and ψ_0_.

It is noted that the p*K*_a_ values used in eq 4 are the values for each single ionizable group without the influence of ionizable/ionized groups on each other. However, the influence the ionizable groups have on each other is introduced by differentiating between surface and bulk pH in relation to the electrostatic surface potential, which is determined by all ionizable groups together as given in eq 5. Therefore, in the equilibrium ionization of the groups as described by eq 4, the surface pH is used.

Now with the electrostatic potential at the surface of a cylinder ψ_0_ known, the electrostatic potential distribution around a cylinder ψ(*r*) was also calculated from the nonlinear Poisson–Boltzmann theory by Stigter^22^ to obtain
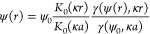
8with γ(ψ(*r*), κ*r*) being a correction factor between the result from the linearized to the full Poisson–Boltzmann equation, i.e., for γ(ψ(*r*), κ*r*) = 1, eq 8 gives the Debye–Hückel result from the linearized Poison–Boltzmann equation. The correction factor γ was systematically calculated and tabulated by Stigter,^22^ and an empirical function was fitted to the tabulated values of γ^22^ to obtain^23^
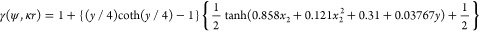
9where *x*_2_ = ^10^log(κ*r*) and *y* = ψ*e*/*kT*. Eq 9 is valid for *y* values up to 8. Using the two equations above, ψ(*r*) can be calculated numerically. Then, the Debye–Hückel curve ψ_DH_(*r*) coinciding with ψ(*r*) from eq 8 at larger *r*, where the potential is smaller than *kT*/*e*, is given by^22,23^
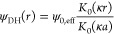
10with
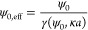
11Thus, to calculate the pair interaction potential between two viruses/cylinders, first, the surface charge and electrostatic surface potential for a single rod at a given pH and ionic strength are calculated from eqs 4–7 as described above. Then, using eqs 9–11, the effective Debye–Hückel potential distribution around a single virus particle can be calculated, which at larger distances from the viruses should correspond to the full potential given in eq 8 and shown in Figure 2 (just a check). Finally, to calculate the interaction potentials between two rods from eq 1 for parallel rods or from eq 3 for tilted rods, only the effective electrostatic surface potential ψ_0,eff_ calculated from eq 11 and the Debye length 1/κ from eq 2 are needed.

Table 1 shows the calculated results for charges, surface potentials, and pair interaction potentials at experimentally relevant values of the ionic strength and at the experimental pH of 7.25. For the (bare) fd virus, there are 2700 major coat proteins, each with five potentially negatively charged and two potentially positively charged groups at the surface, so that the maximum number of net charges theoretically could be 2700 × (−5) = −13 500 charges. This value could be approached theoretically, e.g., at a pH of 13 and an ionic strength of 500 mM, but experimentally, one would first have to determine how long the virus remains intact under such conditions. At the experimental neutral pH, we have a situation that most of the negatively as well as most of the positively charged groups are ionized, resulting in a net charge of roughly (−5 + 2) × 2700 = −8100 elementary charges. This net charge fits especially well at high ionic strength where the surface pH does not become too low, as reflected by eq 5 and the smaller surface potentials at larger ionic strength values.

**Table 1:** Calculated Charges, Potentials, and Pair Interaction Potentials at the Experimental pH of 7.25, *T* = 298 K, ε_r_ = 73 (15% ethanol), *L* = 880 nm, and *a* = 3.3 nm, with Charges and Potentials for Negatively Charged fd and Parallel Pair Interaction Potentials between a Positively and a Negatively Charged Virus Particle with the Same Absolute Charges

I in mM	1/κ in nm	charge in *e*	ψ_0_ in mV	ψ_0,eff_ in mV	*W*_∥_ at *R*_12_ – 2*a* = 3.5 nm in *kT*	*W*_∥_ at *R*_12_ – 2*a* = 5 nm in *kT*
20	2.07	–6995	–101	–84	–724	–328
50	1.31	–7558	–83	–72	–239	–71
100	0.93	–7822	–69	–61	–68	–12.6
200	0.66	–7986	–55	–51	–11.5	–1.09
300	0.54	–8057	–48	–45	–3.0	–0.17
500	0.42	–8132	–39	–38	–0.35	–0.009

The surface pH in combination with the chemical surface equilibrium described in eq 4 gives the surface charge, and from electrostatics, the relation between surface charge and potential is given by eqs 6 and 7. Equations 8–11 result in the effective Debye–Hückel surface potential, which is needed for the calculation of the pair interaction potential that will later be compared to the results from the complex formation.

## Experimental Section

### Materials

Methoxypoly(ethylene glycol) 5000 propionic acid *N*-succinimidyl ester (mPEG-SPA, >80%, molecular mass of the mPEG, 5 kDa), and *N*-ethyl-*N*′-(3-dimethylaminopropyl)carbodiimide hydrochloride (EDAC, >99%) were obtained from Fluka, and absolute ethanol (for analysis) was obtained from Merck. *N*,*N*-Dimethylethylenediamine (DMEDA, >98%) was obtained from Aldrich. Further common chemicals for the preparation of buffer solutions were all of high purity and obtained from Sigma-Aldrich. All solutions containing (modified) fd viruses were prepared in a mixture of 85% water and 15% ethanol (by volume) to prevent microbiological growth.

### Preparation of Chemically Modified fd Viruses

The growth of fd virus and its chemical modification to obtain PEG-grafted viruses (PEG-fd) and positively charged PEG-fd (DMEDA-PEG-fd) were performed following Zhang et al.^15^ with one minor modification. For the PEG grafting, here, a virus concentration of 4 mg mL^–1^ was used instead of the 2 mg mL^–1^ as used by Zhang. After PEG grafting, a small portion of the PEG-fd and fd was dispersed in a 20 mM phosphate buffer with pH 7.5 and with 80 mM NaCl, and the concentration at which the isotropic–nematic phase transition takes place was determined to be 14.3 mg mL^–1^ for PEG-fd and 22.6 mg mL^–1^ for the fd virus without any modification. This clearly indicates a successful PEG grafting (also see Dogic et al.;^13^Figure 2).

**Figure 3:**
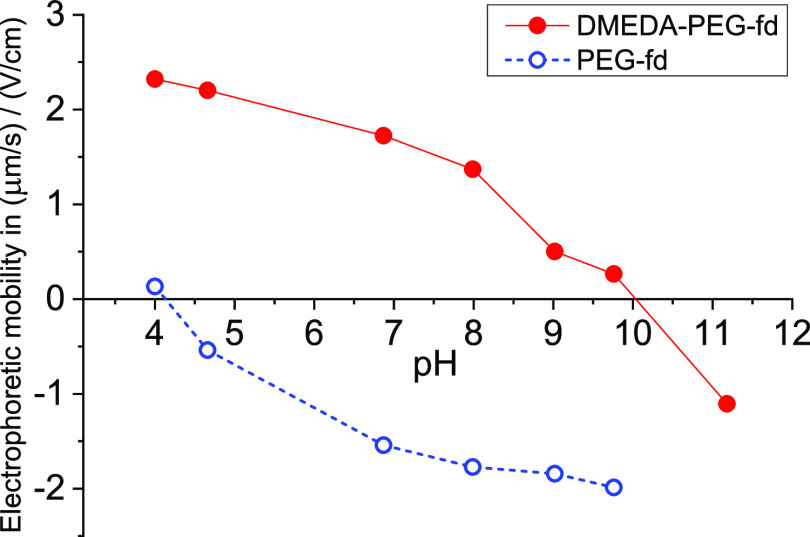
Electrophoretic mobility of PEG-fd and DMEDA-PEG-fd against the pH, as measured in buffer solutions with an ionic strength of 1 mM and containing only monovalent ions.

Then, half of the PEG-fd was chemically modified by *N*,*N*-dimethylethylenediamine (DMEDA) using carbodiimide chemistry, resulting in the majority of the carboxyl groups modified to a tertiary amine group and thereby contributing to a positive charge at neutral pH. After charge reversal, the purified DMEDA-PEG-fd and the PEG-fd were analyzed by electrophoresis. Figure 3 shows the electrophoretic mobility of PEG-fd and DMEDA-PEG-fd as a function of the pH measured for their solutions in buffer solutions with an ionic strength of 1 mM only containing monovalent ions. The experimental results in Figure 3 clearly demonstrate charge reversal around neutral pH.

All virus concentrations were determined from the UV absorption at 269 nm using an absorption coefficient of 3.84 mg^–1^ cm^2^ for the fd virus.^24^ In this way, the concentration of the fd part of PEG-fd is obtained because the PEG was found not to contribute significantly to the absorption at 269 nm.

### Complex Formation

Only 1:1 polyelectrolyte complexes with equal amounts of positively and negatively charged viruses are considered in this study. Complexes were prepared in two ways: (1) directly by mixing equal amounts of the solutions with the same concentrations of positively and negatively charged viruses in imidazole/HCl buffer of pH 7.25 and a contribution to the ionic strength of 1 mM from the buffer, with the total ionic strength of the two solutions adjusted by NaCl; or (2) by starting with a single sample prepared by method 1 followed by diluting the sample in small quantitatively steps and observing the state of the sample after each dilution. As compared to mixing method 1, mixing method 2 requires less sample to obtain a certain number of points in the state diagram. On mixing according to method 1, NaCl concentrations were the same in most cases, but some samples were also formed by mixing positively and negatively charged virus solutions with different NaCl concentrations. We have no indications that this made any significant difference.

### Electrophoresis

The electrophoretic mobilities of PEG-fd and DMEDA-PEG-fd were measured on a Malvern Zetasizer 2000 using the M3 capillary cell. All samples had a total virus concentration of 0.1 mg mL^–1^. To obtain buffer solutions of the required pH and yielding an ionic strength of 1 mM of only monovalent ions, the following buffers were prepared (in order of pH): acetic acid/NaOH, imidazole/HCl, TRIS/HCl, ammonia/HCl, and 1 mM NaOH solution for pH 11. The necessary composition of the buffer was calculated, checking the pH after preparation.

### Polarization Microscopy

Polarization microscopy observations were made on a Zeiss Axioplan 2. The samples were prepared by putting a small drop of sample between the object glass and cover glass, using parafilm as a spacer.

### Static Light Scattering

A series of static light scattering measurements were performed using an ALV/CGS-8F goniometer with a 632.8 nm HeNe laser (ALV, Germany) at 20 °C using vertically polarized light. With this setup, different angles are measured one by one by turning the goniometer. PEG-fd and the DMEDA-PEG-fd stock solutions were cleaned by centrifugation, and the final samples prepared at a concentration of 0.5 mg mL^–1^ were filtered through a nylon syringe filter with 5 μm pore size, directly into cleaned cuvettes with which the light scattering measurements were performed. For all measurements, solvent scattering was subtracted and absolute scattering intensities *R* (Rayleigh ratios) were obtained by normalizing against toluene measurements as standard using *R* as 1.35 × 10^–5^ cm^–1^ for the toluene scattering and corrected for the difference in refractive index between toluene and the samples.

Samples were dispersed in 2 mM imidazole/HCl buffer at a pH of about 7.2 (contribution to the ionic strength 1 mM), with the final ionic strength adjusted with NaCl. First, a sample of PEG-fd and a sample of DMEDA-PEG-fd, both at an ionic strength of 200 mM and a virus concentration of 0.5 mg mL^–1^, were filtrated into cleaned cuvettes and measured separately. Then, these two samples at 200 mM were directly mixed in one of the cuvettes without additional filtration and then measured as a mixture. Further measurements of this mixture at ionic strengths varying from 200 to 500 mM were performed by adding small amounts of solid NaCl directly into the light scattering cuvette. The measurements on the mixed sample at 200, 250, and 300 mM were performed in a time-dependent manner, i.e., at times ranging from 30 min to about 20 h after preparation (salt addition), the scattering intensity at each angle was measured for 1 min at the shorter times after salt addition and for 5 min at longer times after salt addition, but no systematic time dependence could be observed. Therefore, only time-averaged results are given.

## Results and Discussion

The positively charged DMEDA-PEG-fd and the negatively charged PEG-fd were characterized by free solution electrophoresis at an ionic strength (*I*) of 1 mM. The results are shown in Figure 3, demonstrating a successful charge reversal, with an equal absolute charge around neutral pH.

The pH for complex formation was chosen by aiming at approximately equal absolute charges of the positive and negative viruses. Therefore, a pH between 7.1 and 7.25 was adjusted, where 7.25 is the pH with the same absolute value for the electrophoretic mobility and 7.1 is the average of the two isoelectrical points. The pH was set using an imidazole buffer, which only contains monovalent ions. The total ionic strength of the samples was adjusted by adding appropriate amounts of NaCl, with the buffer contributing 1 mM monovalent ions, so that *I* = 1 mM + concentration (NaCl).

**Figure 4:**
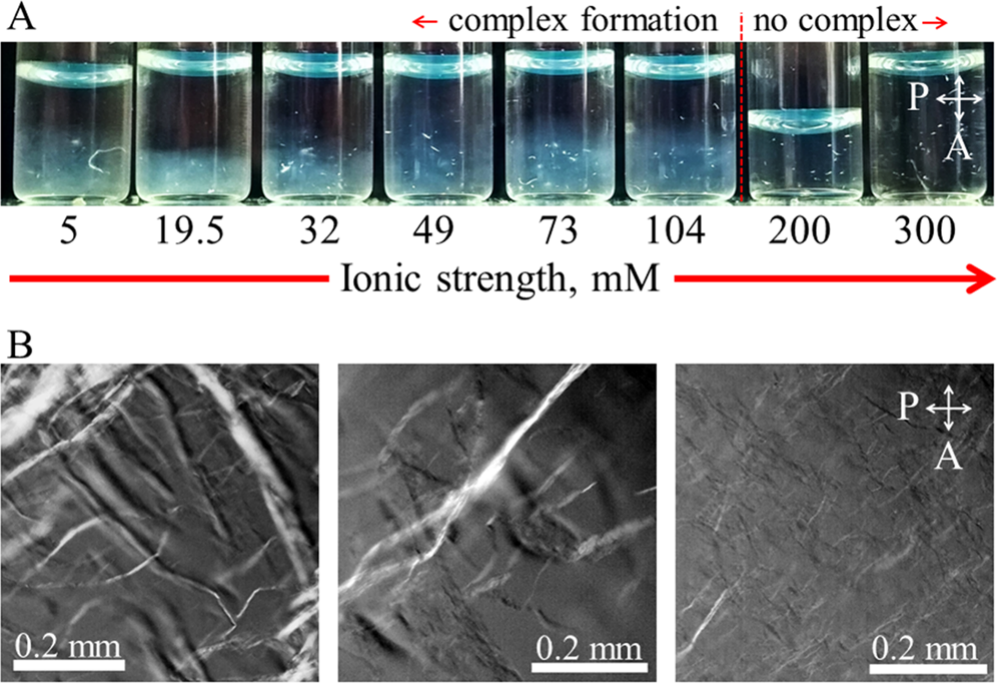
Complex formation at 0.5 mg mL^–1^ fd as a function of the ionic strength *I*. (A) Samples in small bottles between crossed-polarizers, and (B) three samples with *I* values of 5, 32, and 104 mM (from the left to right), observed with the polarization microscope.

Figure 4A shows samples with mixtures of PEG-fd and DMEDA-PEG-fd in a range of salt concentrations corresponding to a range of ionic strength *I* at an fd concentration of 0.5 mg mL^–1^. Complex formation is observed at *I* = 104 mM and below. At *I* = 200 and *I* = 300 mM, visual inspection of the mixed PEG-fd and DMEDA-PEG-fd solutions does not show a difference in the appearance of the pure PEG-fd or DMEDA-PEG-fd samples, i.e., no visual complex formation at these higher *I* is observed. No sharp boundaries are observed between the top and the bottom part of the samples, indicating a flocculated state. Indeed, observations with the polarization microscope as shown in Figure 4B confirm this impression of a flocculated state. In addition, at increasing salt concentration, the flocculated structure appears to become less coarse, until at even higher salt concentration, the visual complexes completely dissolve. Individual fiber-like structures appear to be birefringent, as expected for bundles of oriented fd.

This seems opposed to the observations by Raguzin et al.,^18^ who found scrambled egg structures for the PEC formation from their oppositely charged bottle brush polymers. This different result might be connected to the larger flexibility of the polymer brushes of Raguzin et al., as compared to fd viruses that have an estimated persistence length of 2.2 μm.^12^ Furthermore, our PEG grafting limits direct (close) charge interactions, contrary to the brushes from Raguzin et al., which may also play a role in the apparent different behavior.

**Figure 5:**
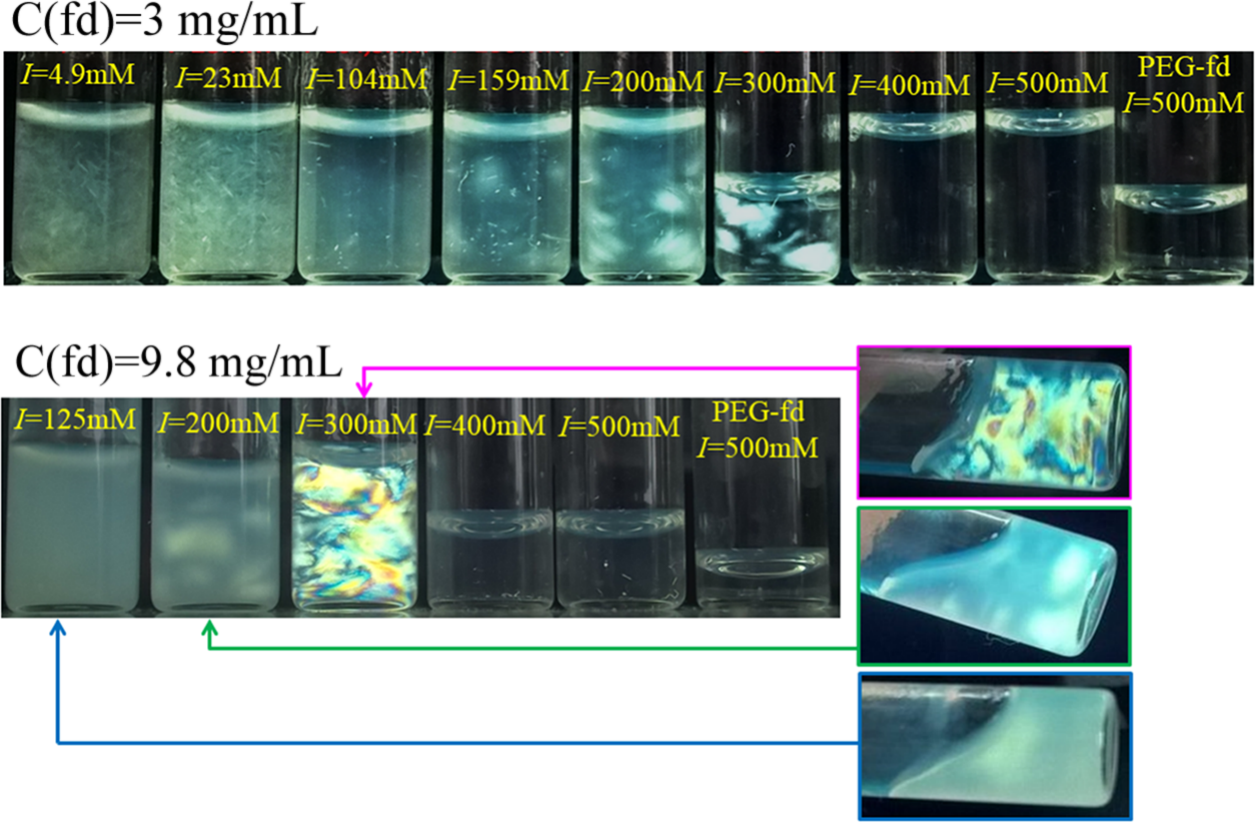
Complex formation at 3 and 9.8 mg mL^–1^ fd as a function of the ionic strength *I*. Samples are in small bottles and shown between crossed-polarizers. For comparison, samples of PEG-fd at 3 and 9.8 mg mL^–1^ are shown. For three samples, the effect of tilting the sample is shown (after about 3–10 s).

At higher fd concentrations of 3 and 9.8 mg mL^–1^ (Figure 5), visual complex formation is already observed at *I* of 300 mM and lower, and in addition, the samples from 159 to 300 mM show a birefringence pattern resembling that of a nematic like liquid crystalline structure (hereafter called nematic like). Apart from these two differences, the trend is similar to the behavior at an fd concentration of 0.5 mg mL^–1^. Tilting the bottles of a few 9.8 mg mL^–1^ samples as shown in Figure 5 illustrates the viscous state of these samples, contrary to the samples without complex formation that are of low viscosity. In agreement with these results, it might be interesting to note that we further observed that a drop of concentrated viscous complex sample did not dissolve in water but could readily be dissolved in a salt solution of 500 mM. Although not further analyzed, this observation indicates reversibility of complex formation. However, we note that this does not demonstrate whether the complexes formed are in a thermodynamic equilibrium state or, e.g., in a long-living nonequilibrium gel state. This also holds for the observation that the appearance of the samples shown in Figure 5 remained almost the same over months.

**Figure 6:**
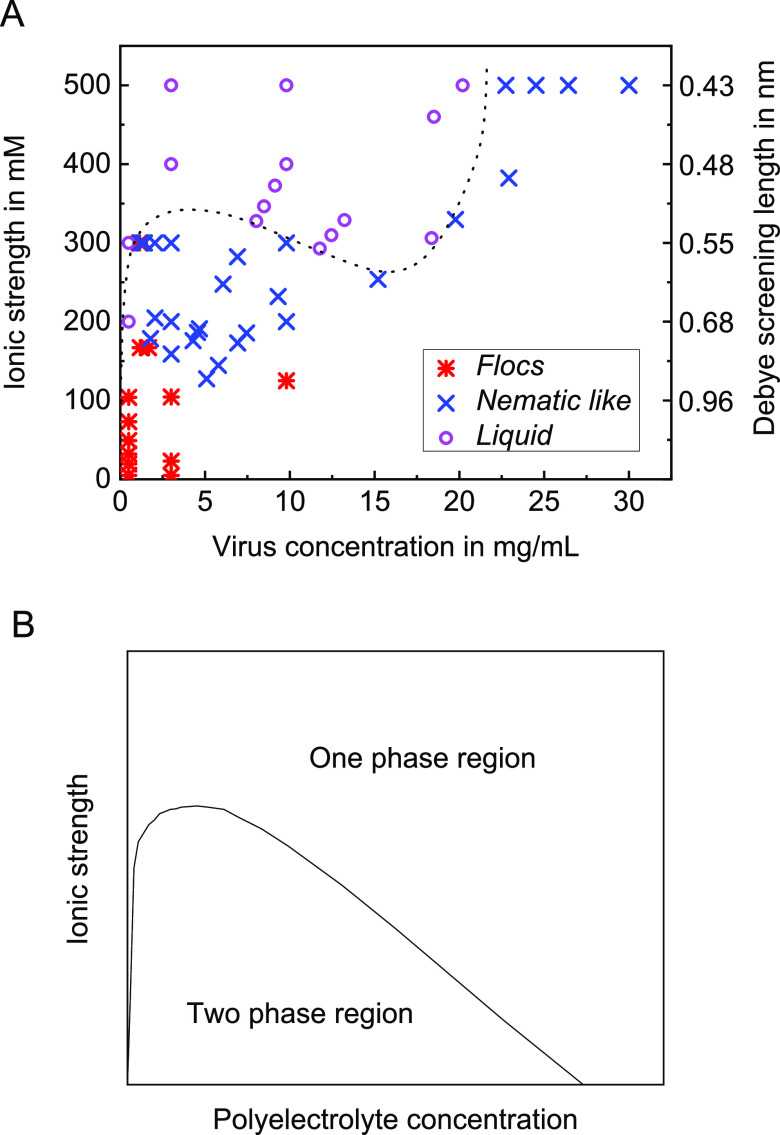
(A) State diagram of complex formation as visually observed between crossed-polarizers. The liquid phase denotes samples without clear visible complex formation, i.e., samples looking like the samples with an ionic strength of 400 or 500 mM in Figure 5. The dotted line is a guide to the eye, separating the region where complex formation occurs from the liquid state region without visual complex formation. More information is given in the main text. (B) Sketched phase diagram of a typical binary phase separation.^6,9^ Here, the one-phase region from Figure 6B corresponds to the liquid phase in Figure 6A.

A state diagram of the complex formation as a function of virus concentration and ionic strength as visually observed between crossed-polarizers is shown in Figure 6. Three states as presented in Figures 4 and 5 are observed: (1) flocs; (2) nematic like, without a flocs-like appearance; and (3) liquid, a clear liquid of low viscosity without clear visible signs of complex formation. We note that the change from the “nematic like” to the “flocs” state is a gradual transition for which no really clear boundary can be identified; nevertheless, it seems to reflect the transition between two states with a different appearance. The curved dotted line in Figure 6 is a guide for the eye and denotes the border between the liquid state and the state where visual complex formation is observed. It is interesting that the part of the state diagram below a virus concentration of 12–15 mg mL^–1^ resembles the diagram of a typical phase separation into a dilute and a concentrated polyelectrolyte phase as sketched in Figure 6B, apart from the fact that here only a flocs or a birefringent state is observed instead of an equilibrium phase separation. However, at virus concentrations above 15 mg mL^–1^, the appearance in Figure 6A is completely different from that in Figure 6B and the dotted line starts turning up. This behavior can be understood if one realizes that at 500 mM the electrical double layer with a thickness of around 0.5 nm probably lies within the PEG layers and that dispersions of viruses without modifications, i.e., pure repulsive particles, also form a phase transition to a liquid crystalline (nematic) phase.^13^ It is probably this behavior that is observed for different virus concentrations at 500 mM salt concentration, whereas at lower salt concentrations, the effective attraction between the oppositely charged viruses increases and visual complex formation occurs. Therefore, the state diagram shows a transition from a repulsion-induced formation of a nematic like phase around 20–23 mg mL^–1^ fd and *I* of 400–500 mM to an attraction-induced nematic like phase at virus concentrations below 15 mg mL^–1^ and *I* of 300 mM or lower.

In connection to this behavior, in Figure 6A, we observe an interesting fact on performing a dilution series starting at 30 mg mL^–1^ virus and *I* of 500 mM and going in the direction of 0 mg mL^–1^ virus and 1 mM using a buffer solution without salt and without viruses for the dilution. Following this dilution line, between about 15 and 20 mg mL^–1^, a re-entrance behavior of the nematic like state is observed. It might be speculated that the re-entrance behavior is a result of the gradual transition between the repulsion-induced and the attraction-induced birefringent state as discussed above.

A phase diagram of oppositely charged colloidal spheres has been calculated theoretically,^25^ but we are not aware of similar calculations for oppositely charged cylindrical objects.

We also consider the possibility of characterizing the existence of soluble aggregation or other structures with static light scattering. The fd virus is too large to reach the limit of small scattering angles θ, from which the absolute molecular weight *M*_w_ of possible aggregates could be determined. However, if a cylindrical shape of the aggregates is assumed, the molecular weight per unit of rod length (*M*_w_/*L*) might be obtained from a so-called Holtzer^26^ plot. If for a dilute solution of cylindrical particles, *QR*/(*Kc*) is plotted against the scattering vector amplitude *Q*
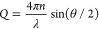
12with *n* being the refractive index of the sample and λ being the wavelength of the light, then the high *Q* limit is constant and is equal to π*M*_w_/*L*, with *c* being the concentration of the scattering component, *R* being the Rayleigh ratio for vertically polarized light, and
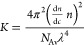
13with d*n*/d*c* being the refractive index increment and *N*_Av_ being the Avogadro constant.

In this way, a measure for the size of possible aggregates might be obtained, where it is noted that the result is only sensitive to the quotient *M*_w_/*L*, i.e., a change in diameter is observed but not a change in length. Still, for small aggregates, it might be a reasonable assumption that the length of the aggregate is equal to the length of the virus, so that *M*_w_ can be obtained. Of course, an exact quantitative measure for possible aggregation is only obtained for a parallel aggregation with a constant diameter over the length of the aggregate. Moreover, in practice, aggregates might be polydisperse and an average increase in diameter will be obtained. Furthermore, we note that this analysis can only be applied for virus concentrations that are low enough such that it is reasonable to neglect excluded volume effects.

**Figure 7:**
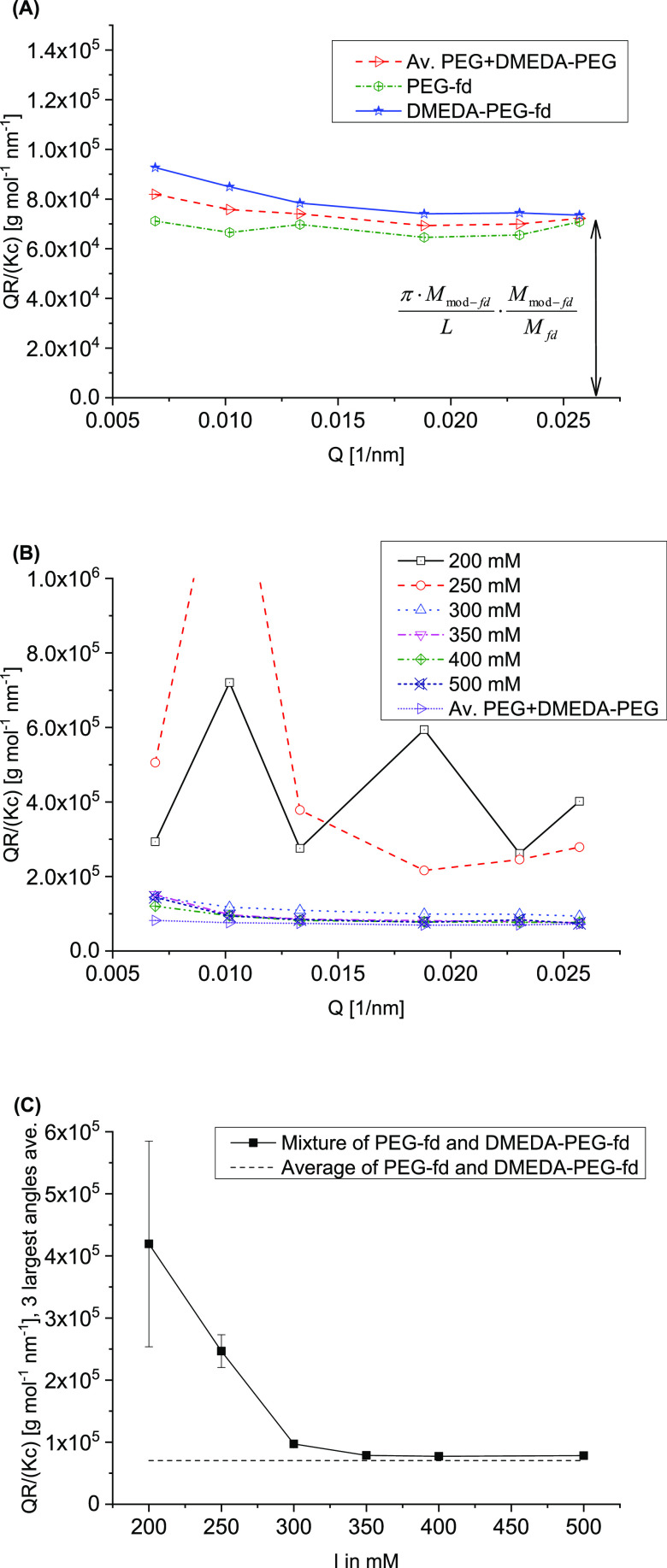
Static light scattering results at *c* of 0.5 mg mL^–1^: (A) Holtzer plots for PEG-fd and DMEDA-PEG-fd at *I* of 200 mM with average; (B) Holtzer plots of the mixture of PEG-fd and DMEDA-PEG-fd at *I* of 200–500 mM; (C) average of *QR*/(*Kc*) of the three largest scattering vectors *Q* from (B) plotted against *I* and compared to the average of PEG-fd and DMEDA-PEG-fd at 200 mM (before mixing). The error bars give 2 times the standard deviation; for *I* of 300 mM or larger, the estimated errors are smaller than the symbols.

First, a solution of PEG-fd and a solution of DMEDA-PEG-fd at 0.5 mg mL^–1^ were measured separately, the results of which are shown in Figure 7A. As used throughout this study, the concentration of 0.5 mg mL^–1^, as determined from the UV absorption, is the concentration of the virus part of the modified fd (mod-fd, average of PEG-fd and DMEDA-PEG-fd), so the concentration used is too low and thereby *QR*/*Kc* is too large by a correction factor (*M*_mod-fd_/*M*_fd_) as denoted in Figure 7A.

An estimate for the refractive index increment of the modified fd virus (d*n*/d*c*)_mod-fd_ is connected to the result for *M*_mod-fd_/*M*_fd_, and its calculation is given in the following paragraph. Starting with an estimate for (d*n*/d*c*)_mod-fd_ = (d*n*/d*c*)_fd_ = 0.174 mL g^–1^ (ref (27), extrapolated to 632.8 nm) and using *L* of 880 nm and *M*_fd_ of 16.4 × 10^6^ g mol^–1^ from ref (28), we obtain *M*_mod-fd_/*M*_fd_ = 1.075. Now, the refractive index increment of PEG-fd is estimated from (d*n*/d*c*)_PEG-fd_ = *w*_fd_(d*n*/d*c*)_fd_ + *w*_PEG_(d*n*/d*c*)_PEG_ with *w*_fd_ = *M*_fd_/*M*_PEG-fd_ and *w*_PEG_ = 1 – *w*_fd_ = 1 – *M*_fd_/*M*_PEG-fd_, which are the weight fractions of fd and PEG in PEG-fd respectively, and (d*n*/d*c*)_fd_ and (d*n*/d*c*)_PEG_ are the corresponding refractive index increments. The value of (d*n*/d*c*)_PEG_ is 0.134 mL g^–1^ at 632.8 nm as obtained from the literature.^29^ Now, with the first estimate for *M*_mod-fd_/*M*_fd_ = 1.075 given above, a new value for (d*n*/d*c*)_PEG-fd_ can be calculated, and then, the new estimate for (d*n*/d*c*)_PEG-fd_ can be used to calculate a new value for *M*_PEG-fd_/*M*_fd_ again, and repeating this procedure a few times results in constant values, i.e., iterating to the final result, obtaining (d*n*/d*c*)_PEG-fd_ = 1.170(4) mL g^–1^ and *M*_PEG-fd_/*M*_fd_ = 1.099. Then, considering the moderate difference between the refractive index increments of PEG-fd and fd and considering the structural similarity between bound DMEDA and PEG, it seems reasonable to assume that the contribution to the refractive index increment of bound PEG and DMEDA is not too different, and therefore, it seems reasonable to approximate that (d*n*/d*c*)_mod-fd_ = (d*n*/d*c*)_PEG-fd_ and *M*_mod-fd_/*M*_fd_ = 1.099, which, considering the above-mentioned approximations, should only be taken as an approximate value.

The moderate increase in scattering intensity for DMEDA-PEG-fd as compared to PEG-fd may be partly attributed to the additional chemical modification but could also indicate a tiny amount of dust in the sample, which would also explain the slightly increasing intensity for DMEDA-PEG-fd at lower *Q* as compared to PEG-fd. Furthermore, the variation in *QR*/(*Kc*) as a function of *Q* for PEG-fd seems to reflect the accuracy of the measurement. Nevertheless, for the largest *Q* values, an almost constant *QR*/(*Kc*) is found, and the fact that a reasonable value for *M*_mod-fd_/*M*_fd_ is found is an indication that the analysis using Holtzer plots is feasible. Assuming that the ratio *M*_mod-fd_/*M*_fd_ = 1.099 has to be attributed mainly to PEG grafting, a rough estimate of about 325 PEG molecules per virus can be given.

After measuring the solution of PEG-fd and the solution of DMEDA-PEG-fd separately, these two samples were mixed in one of the cuvettes and measured at increasing ionic strengths by repeated addition of solid NaCl to the same cuvette. Figure 7B gives the Holtzer plots for these mixtures at a *c* of 0.5 mg mL^–1^ and an ionic strength ranging from 200 to 500 mM, compared to the average of the individual PEG-fd and DMEDA-PEG-fd scattering before mixing as given in Figure 7A. Although no clear visual complex formation is observed for these compositions (see Figure 4A), for a part of the samples, clear effects are observed in the light scattering results. Especially for 200 and 250 mM, the scattering intensities are strongly increased as well as strongly varying with *Q*, also at larger *Q*. Compared to these two ionic strengths, only a weak increase in intensity is observed for 300 mM, whereas for 350–500 mM, only a tiny increase at high *Q* is found, which might be attributed to a tiny amount of additional dust resulting from mixing PEG-fd and DMEDA-PEG-fd as well as the salt additions.

In Figure 7C, the results of the three largest *Q* values in Figure 7B are averaged, summarizing the results from Figure 7B. The origin of the increase and variation in light scattering intensity at 200 and 250 mM is not clear—it may be aggregates, small nematic/cholesteric droplets, or domains with different densities or orientations; however, to confirm the origin of these structures, more measurements would be needed, which is outside the scope of the present paper. However, the observation that the intensity increase and strong variations with *Q* at 200 and 250 mM salt disappear completely after adding additional salt (Figure 7B) indicates the reversibility of the “structures” formed. In addition, the fact that these “structures” reversibly dissolved in the same cuvette and same sample, just by adding some salt and mixing, shows that the undulations and the peak cannot result from experimental artifacts like dust or scattering from tiny scratches on the cuvette, or something similar, but result from the sample. Finally, considering the 300 mM results, the static light scattering measurements indicate that (semi-)quantitative results can be obtained from static light scattering using a Holtzer plot analysis.

**Figure 8:**
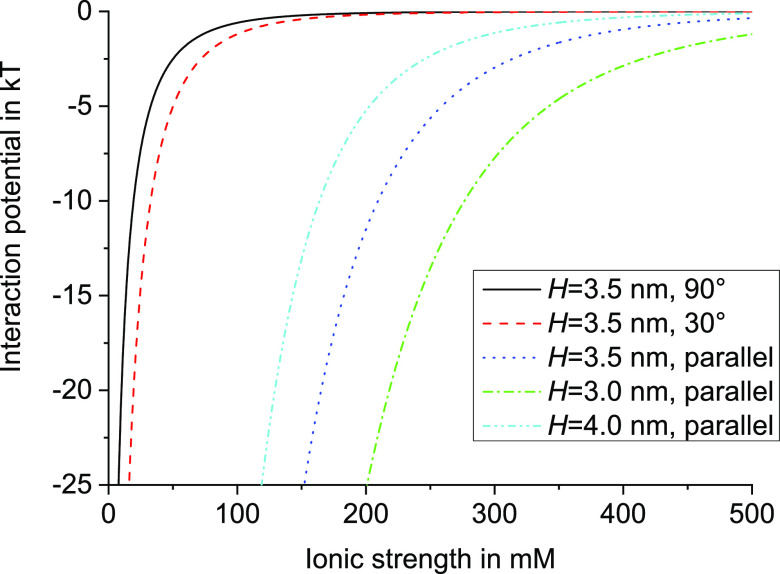
Electrostatic pair interaction potential between two oppositely charged viruses as a function of the ionic strength, at pH 7.25, at the closest surface-to-surface distances *H* of 3.0, 3.5, and 4.0 nm and at three different angles, calculated including charge regulation following eqs 1–11.

To put the observed results from light scattering and visual complex formation on a more solid basis, calculations are performed on the pair interaction potential between two oppositely charged viruses, modeled as oppositely charged cylinders, as described above in eqs 1–11. Results of these calculations are shown in Figure 8 for three different angles between the cylinders. As expected, the attraction between oppositely charged viruses is much stronger for parallel viruses than for tilted viruses. This means that the parallel orientation of the viruses is favored, which appears to agree with the birefringent states found and also with the elongated shape of the aggregates found for the flocculated states in Figure 4B.

Within the calculations, the closest distance between the surfaces of the viruses *H* represents the effect of the PEG steric stabilization layer and is the only adjustable parameter. A first estimate of *H* was obtained by adjusting *H* in steps of 0.5 nm to obtain an interaction potential at distance *H* that fits the experiments. For that, the result at 300 mM ionic strength is used, for which the interaction potential is expected to have a value on the order of several *kT*, because within the state diagram (Figure 6A), 300 mM is just low enough to observe macroscopic visual PEC formation at 3 mg mL^–1^ virus concentration, but no visual PEC formation is observed at a virus concentration of 0.5 mg mL^–1^. As can be obtained from Figure 8, at 300 mM, the interaction potentials at *H* of 3.0, 3.5, and 4.0 nm are −7.7, −3.0, and −1.1 *kT*. Here, −1.1 *kT* seems too small to induce macroscopic PEC formation, but both −3.0 and −7.7 *kT* cannot be excluded. However, for 200 mM and *H* = 3.0 nm, an interaction potential of −25 *kT* is found, which seems a bit too high to explain that no macroscopic PEC formation is observed at a virus concentration of 0.5 mg mL^–1^. In summary, *H* in between 3.0 and 3.5 nm might be possible, but *H* = 3.5 nm seems more likely. It is noted that this value for *H* of 3.5 nm is only a rough estimate.

However, *H* can also be estimated from the concentration of the isotropic–nematic (I–N) transition at 500 mM. At this ionic strength, the interaction between oppositely charged viruses is expected to be mainly repulsive, i.e., dominated by the steric stabilization of the grafted PEG layers, resulting in an effective diameter of the viruses *D*_eff_. It has been suggested that there is a relationship between *D*_eff_ and the concentration of the virus at the I–N transition, given by *c* (in mg mL^–1^) = 222/*D*_eff_ (in nm).^13^ For a concentration at the I–N transition at 500 mM of 21.5 mg mL^–1^, this results in an estimate for the effective diameter of 10.3 nm. As *D*_eff_ = 2*a* + *H* and *a* = 3.3 nm, this gives a value of *H* = 3.7 nm, which is in good agreement with the estimate of *H* = 3.5 nm (or slightly lower) given above.

**Figure 9:**
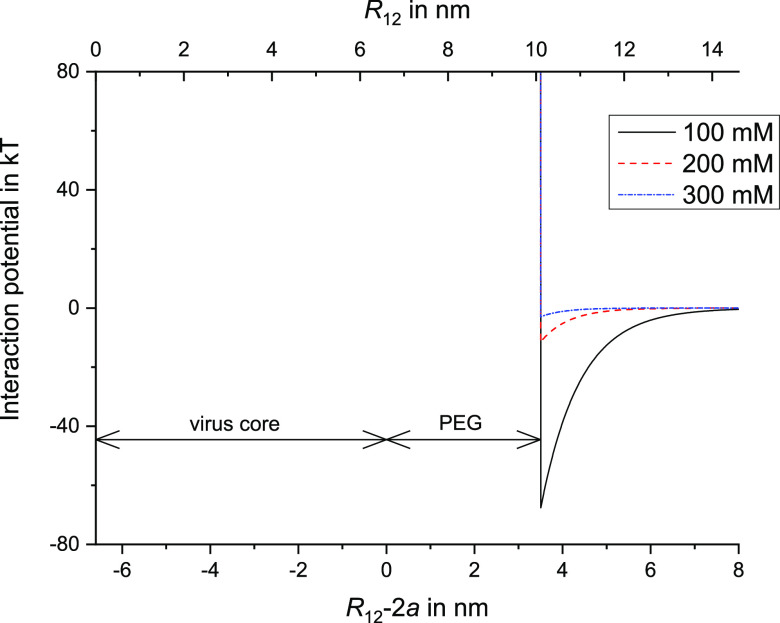
Pair interaction potential against separation of two parallel viruses for ionic strengths of 100, 200, and 300 mM, at a pH of 7.25 and approximating the effect of the PEG layer as a hard-cylinder repulsion. Note that the maximum depth of these potentials can be read from the curve for parallel viruses in Figure 8 at the corresponding ionic strength.

Thus, if we assume a hard-cylinder repulsion at the distance between the center of axis of the viruses *R*_12_ = 2*a* + *H* = *D*_eff_ with *H* = 3.5 nm, an estimate of the full interaction potential as a function of the separation of the viruses can be calculated, which is shown in Figure 9 for parallel rods and ionic strengths of 100, 200, and 300 mM.

Indeed, if we compare the interaction potentials at different ionic strengths to the complex formation as visually observed, clear homogeneous samples are observed for *I* of 400 and 500 mM corresponding to a low attraction (less than for 300 mM), whereas the flocculated structure is found for *I* of 100 mM or lower, corresponding to a strong attraction between oppositely charged viruses. Furthermore, for the intermediate values of *I* of 200–300 mM, corresponding to moderate attractions between the viruses, birefringent samples are found resembling a nematic phase. In addition, the moderate attraction at 300 mM also fits the corresponding static light scattering results as discussed above (see Figure 7C).

Note that the interaction potentials in Figure 9 appear quite narrow compared to the diameter of the virus with PEG. Together with the depth of the attractive pair potential, this could explain the formation of nonequilibrium gel-like structures.^30^ Also, the results from Duschner et al.^17^ on PEC formation between oppositely charged brush polymers show a kinetically controlled PEC formation for a highly charged brush polymer, contrary to topologically controlled PEC formation for a slightly charged brush polymer. We further note that the mechanism of aggregation and phase separation in our systems is different from an interesting mechanism recently discussed for high polyelectrolyte concentration and site-specific intrinsic ion pair formation between individual positive and negative groups of the polyelectrolytes.^31^ These intrinsic ion pairs are not possible in our system because of the grafted PEG, which leads inevitably to a mean field type of charge interaction in the present case.

For our colloidal type of polyelectrolytes, a description of the (mean field) pair interaction potential is an important step toward understanding the formation of either equilibrium liquid states or glassy or gel-like “solid” states, as well as other properties. In summary, the various experimentally observed states, as well as the static light scattering results, correlate well with calculations of the interacting potential between two oppositely charged fd viruses.

## Conclusions

We present an explorative study on the formation of polyelectrolyte complexes from chemically modified fd viruses with steric repulsion and electrostatic attraction. Depending on the virus concentration and ionic strength, birefringent states resembling a nematic phase were observed along with flocculated states with increased turbidity and clear solutions without visible signs of polyelectrolyte complex formation. A state diagram summarizing the experimental observations is given. In some samples without, but approaching visually observable, complex formation, a strongly varying increase in static light scattering intensity could be observed. The origin of these results is not clear—they might be explained by aggregates, small nematic/cholesteric droplets, or domains with different densities or orientations. Nevertheless, the light scattering results could also aid in future studies of soluble polyelectrolyte complexes.

It is shown that the results from light scattering as well as visual complex formation can be rationalized by comparison to calculations modeling the attractive electrostatic pair-interactions between viruses as two oppositely charged cylinders. Importantly, the only adjustable parameter, the distance of closest approach resulting from the PEG layers of 3.5 nm, could be estimated straightforwardly from a single measurement of the isotropic–nematic phase transition concentration at *I* of 500 mM, where interactions seem to be dominated by the repulsion between the PEG layers. Hence, one single transition in the state diagram gives an estimate of the only adjustable parameter for the calculation of the interaction potential.

As expected, the calculated attraction between oppositely charged viruses increases for decreasing salt concentrations. Generally, for strong attractions (low salt), flocculated states are observed, whereas for moderate and low attractions, nematic like states may be observed. The fact that no liquid complexes (coacervates) are observed might be connected to the relatively narrow attractive pair interaction potentials found in the model calculations.^30^ Here, a comparison to similarly designed polymeric bottle brush systems might also be of interest, especially if for these systems an interaction potential can be described too.

In summary, because of the possibility to theoretically model the pair interaction potential between oppositely charged viruses and the possibility of light scattering measurements to observe small soluble structures, these chemically modified fd viruses might be useful for further interesting (semi)quantitative model studies. Concerning the present study, it might be of interest if the results from the state diagram could be compared to theoretical or simulation results. As the most important parameters defining the system are known, and specific effects from charge–charge interactions are limited and therefore generic results are expected, a critical comparison might be possible. In addition, in the future, an improved interaction potential might be obtained by quantitatively describing the repulsive interaction potential by the grafted polymers, as was done by Witten and Pincus^32^ for spherical polymer brushes and successfully used, e.g., to describe the interactions between block copolymer micelles.^33^ As far as we know, no such description has yet been derived for cylindrical particles.
